# Supplementary material for: Linking Circulating Serum Proteins with Clinical Outcomes in Esophageal Adenocarcinoma—An Emerging Role for Chemokines
Source: Cancers (Basel). 2020 Nov 13;12(11):3356. doi: 10.3390/cancers12113356 (PMC7698106; doi:10.3390/cancers12113356)
Supplement: Supplementary file 1 [file cancers-12-03356-s001.pdf]

Article

Linking Circulating Serum Proteins with Clinical Outcomes in Esophageal Adenocarcinoma—An Emerging Role for Chemokines

Noel E Donlon, Andrew Sheppard, Maria Davern, Fiona O'Connell, James J Phelan, Robert Power, Timothy Nugent, Kate Dinneen, John Aird, John Greene, Paul Nevins Selvadurai, Anshul Bhardwaj, Emma K Foley, Narayanasamy Ravi, Claire L Donohoe, John V Reynolds, Joanne Lysaght, Jacintha O'Sullivan and Margaret R Dunne

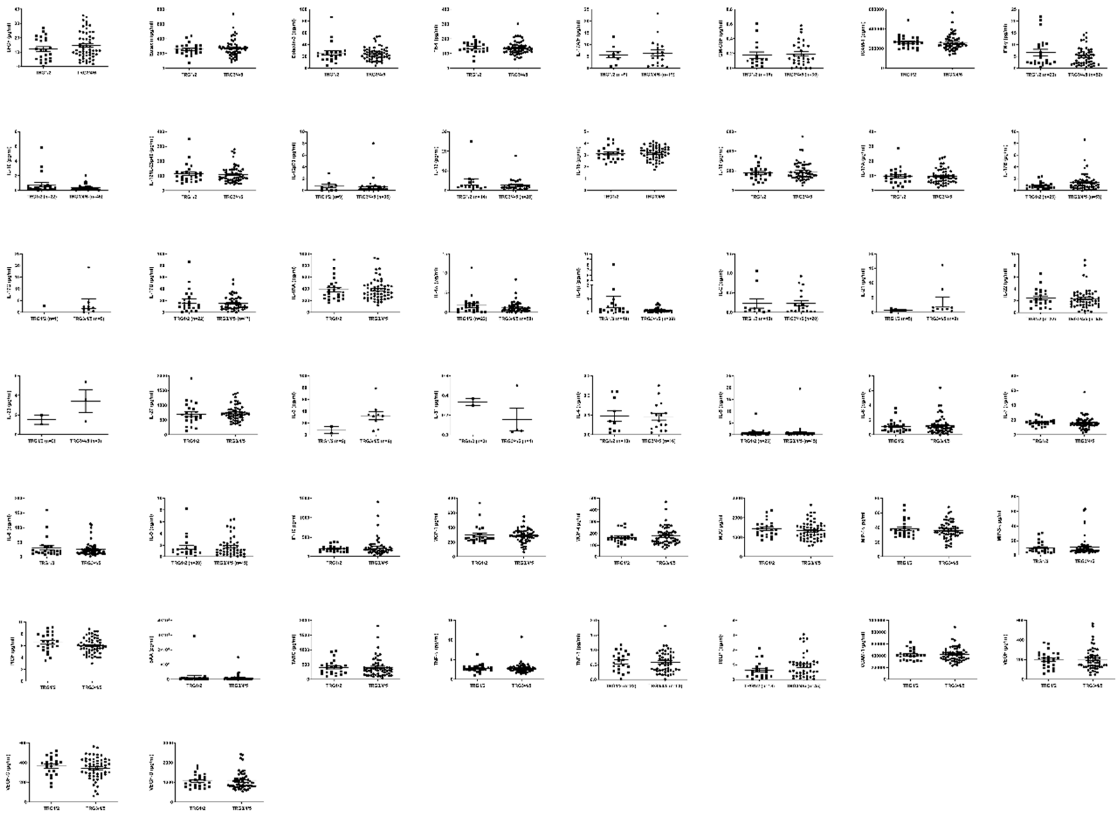

Figure S1. No difference in levels of serum proteins was observed between low and high TRG groups.

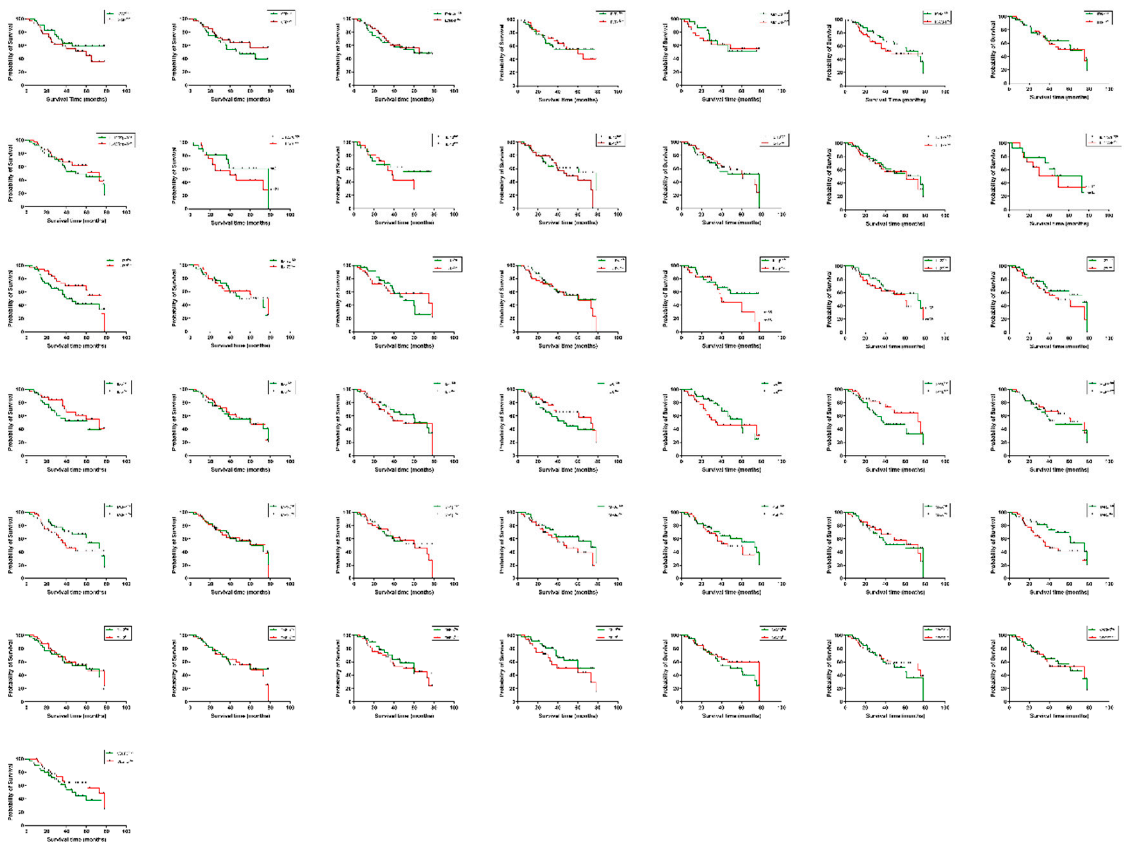

Figure S2. Serum proteins were not significantly associated with OS time.

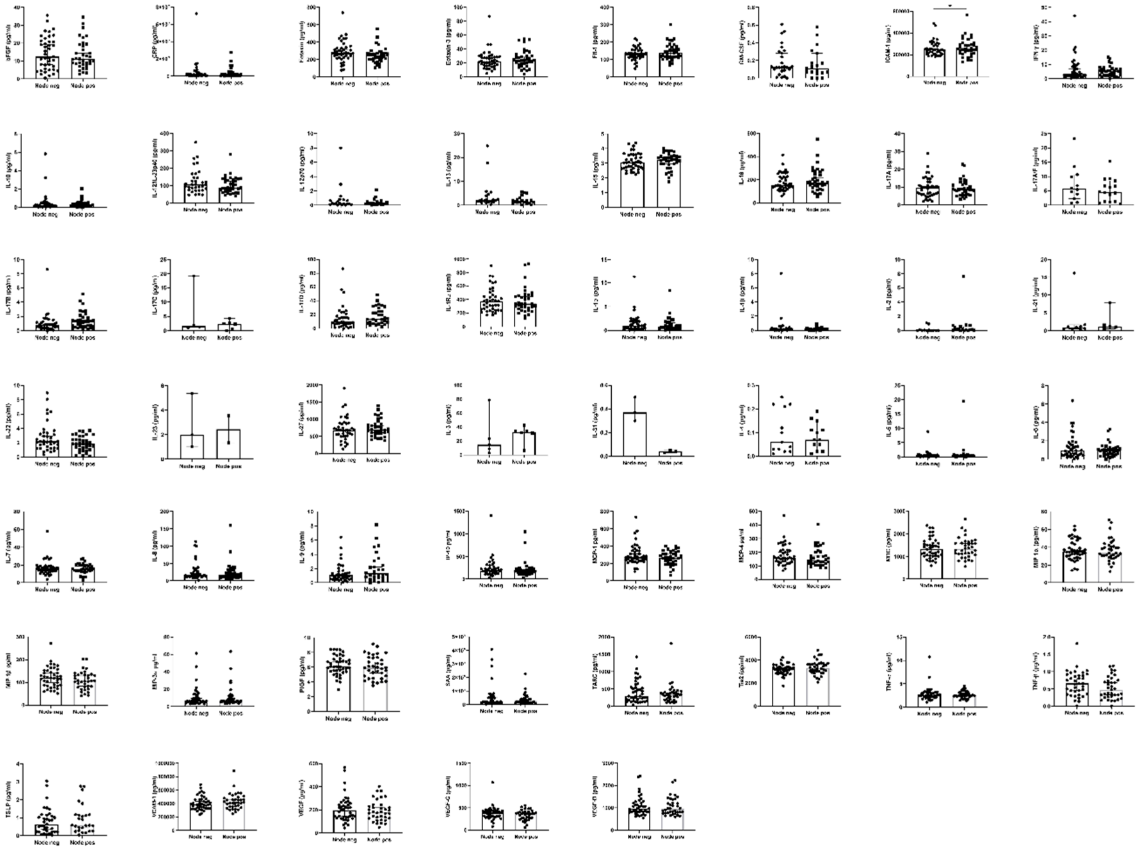

Figure S3. Serum protein levels were not altered between node negative and node positive groups.

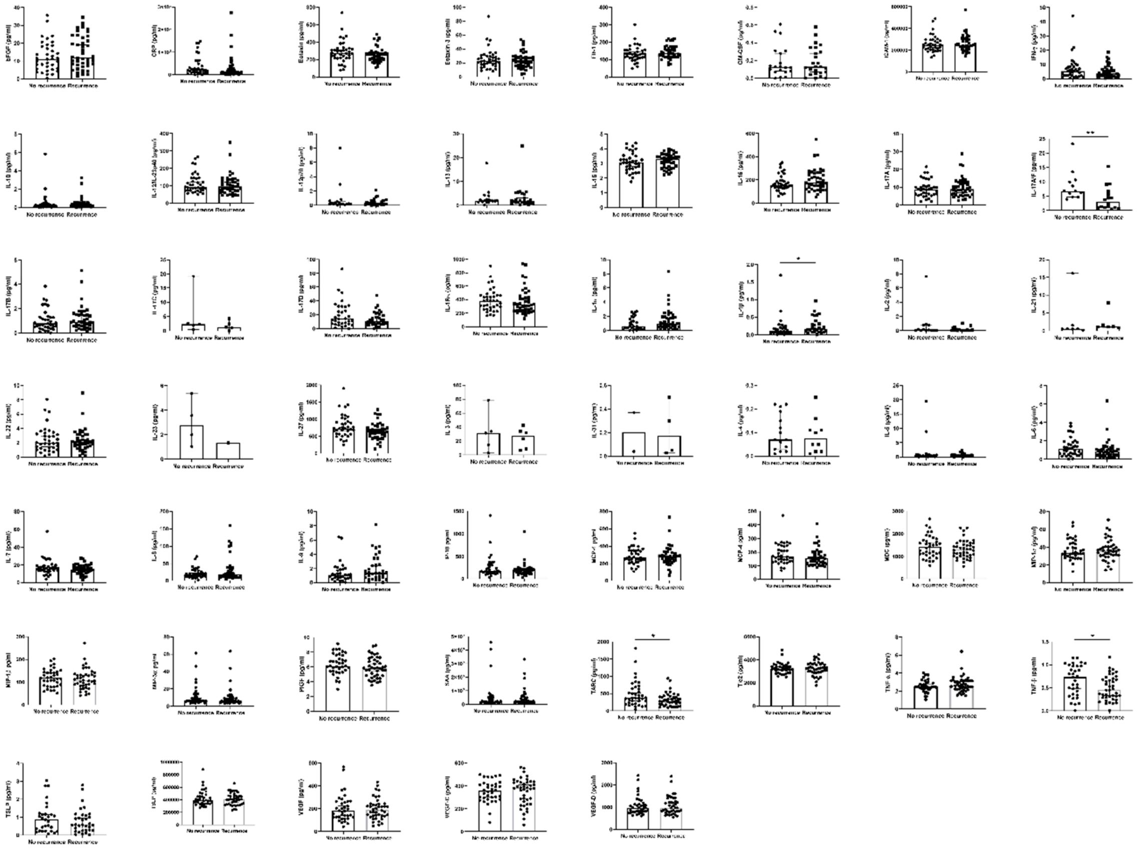

Figure S4. Serum protein levels were not altered between recurrence and no recurrence groups.

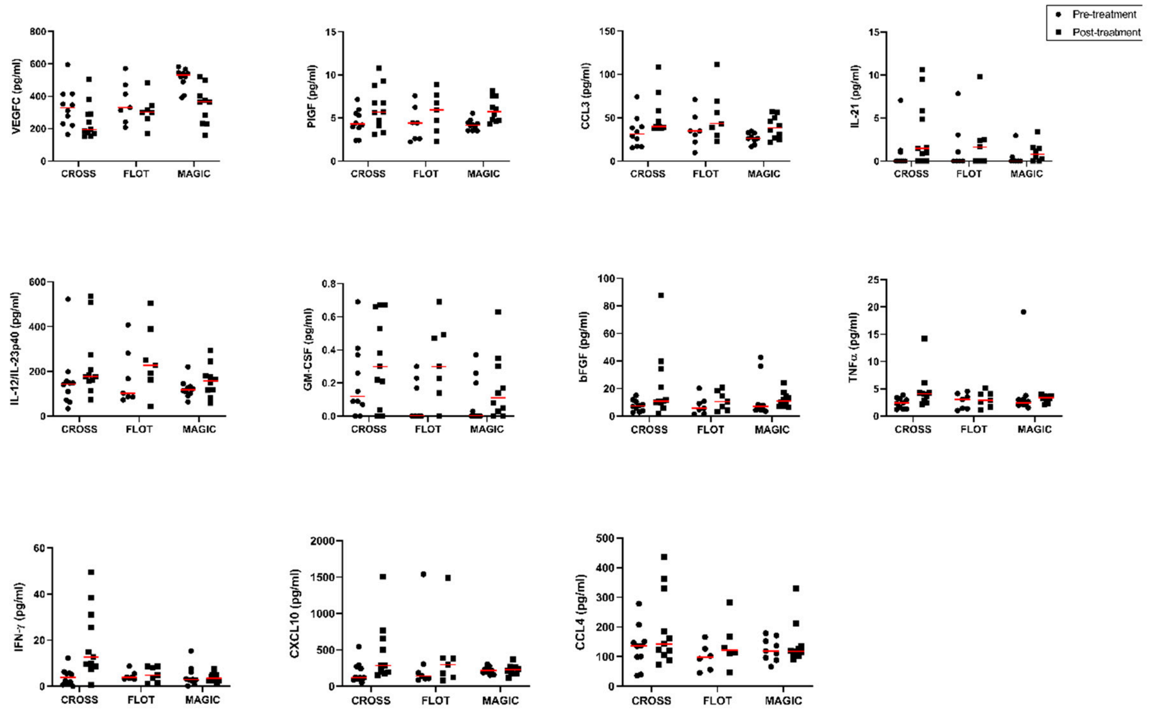

Figure S5. Levels of serum marker expression before and after neo-CT treatments.

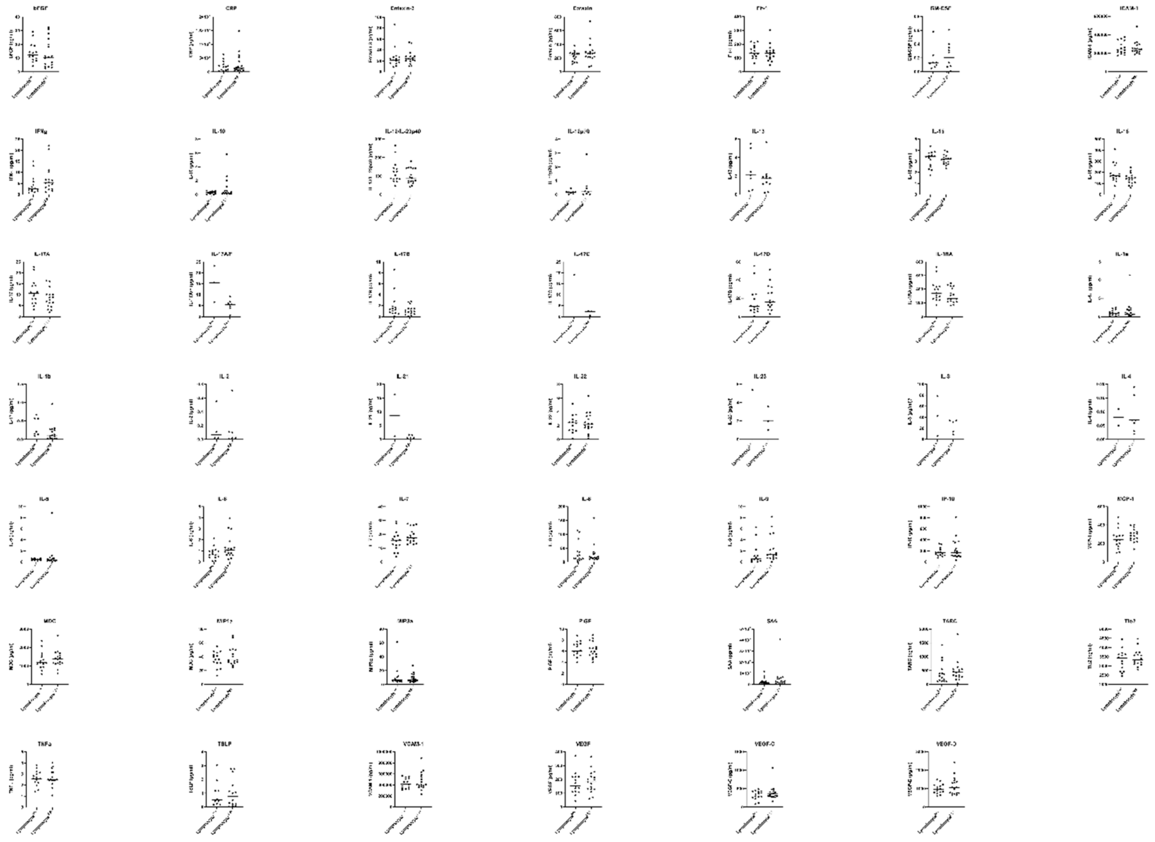

Figure S6. Serum protein levels were not altered between lymphocyte high, and low tumor groups.
